# Sinking city: A multidimensional dataset for urban and land subsidence modelling based on satellite imagery in Jakarta, Indonesia (2016-2024)

**DOI:** 10.1016/j.dib.2026.113048

**Published:** 2026-07-02

**Authors:** Zahratu Shabrina, Fajrun Wahidil Muharram, Muhammad Asa, Dekka Dhirgantara Putra, Jin Rui

**Affiliations:** a Department of Geography, King’s College London, WC2R 2LS, UK; b Department of Geography, University College London, WC1E 6BT, UK; c Resilience Development Initiative, Bandung, West Java, 40123, Indonesia

**Keywords:** Land subsidence, Urban morphology, Impervious surface, Residential area

## Abstract

Jakarta, the world’s fastest-sinking city, faces complex urban challenges from the complexity of its urban morphology, infrastructure, and environmental conditions. This study presents a descriptor for a multidimensional database of Jakarta, Indonesia, that can be used to analyse the city’s subsidence and understand the frequent flooding events throughout the city. The data comprise four different dataset modelled using satellite imagery: (1) land subsidence modelling to quantify subsidence rates in Jakarta based on Sentinel-1 SAR data processed using the open-source package LiCSBAS (2) spectral indices to highlight vegetation and built-up areas, namely the Normalised Difference Vegetation Index (NDVI) and the Normalised Difference Building Index (NDBI) using Landsat 5, Landsat 7, and Landsat 9 imagery, and urban morphology datasets, including (3) impervious surface areas using Sentinel-2 based on adaptation of the Enhanced Normalised Difference Impervious Surface Index (ENDISI) formulation and (4) the proportion of residential areas modelled using K-means clustering. Data processing was performed using Google Earth Engine (GEE) and Python to generate a critical dataset to support analysis and modelling of urban resilience, climate adaptation, and mitigation towards flooding in the context of sinking city. Robust technical validation techniques were performed to ensure data accuracy and validity, with detailed limitations presented. The result presents a series of modelled datasets that can inform further urban analysis and modelling related to flooding events in Jakarta, with a methodology that can be replicated globally, especially in other Global South cities.

Specifications Table

**Table:** 

Subject	Earth & Environmental Sciences
Specific subject area	Geospatial dataset for understanding sinking Jakarta for flood modelling (land subsidence, NDVI, NDBI, impervious surface and proportions of residential areas)
Type of data	The dataset is provided in three formats: raster (TIFF), vector (shapefile) and tabular (CSV)
Data collection	This dataset present a multi-source geospatial dataset through integrating remote sensing and building footprint data spanning the period of 2016–2024. These datasets are compiled and modelled to support the analysis of urban flood risk drivers in Jakarta and can be replicated for other sinking megacities globally. The land subsidence rate modelling was derived from Copernicus Land Monitoring Service which provides satellite-based surface deformation estimates applied to Sentinel-1 synthetic aperture radar imagery and processed end-to-end with the open-source package LiCSBAS. Vegetation and built-up dynamics were characterised through the Normalised Difference Vegetation Index (NDVI) and Normalised Difference Built-up Index (NDBI), both computed from Landsat surface reflectance imagery for the period 2015–2024 using threshold-based rules. The impervious surface area (ISA) layer for 2016–2024 was produced from Sentinel-2 multispectral imagery using supervised classification approaches. The proportion of residential area for 2023 was derived from four spatial data sources: building height information from the Google Open Buildings 2.5D Temporal Dataset, RW *(Rukun Warga)* administrative unit boundaries defining residential block, NDVI-based estimates of green space and farmland coverage and building footprint geometry used to characterise density and layout. Residential classification followed the typological framework which distinguishes four settlement types: (1) high-rise, (2) regular, (3) urban village, and (4) rural, useful in providing a socio-spatial layer that contextualises flood exposure within distinct settlement typologies. All datasets were validated for accuracy and geometric consistency in Jakarta. The resulting multi-layered dataset is made openly available to support reproducibility and future urban flood risk research.
Data source location	Study area: Jakarta, Indonesia
Data accessibility	Repository name: Zenodo Data identification number: 10.5281/zenodo.19456870 Direct URL to data: https://doi.org/10.5281/zenodo.19456870
Related research article	None

## 1 Value of the Data

• The dataset presents a publicly available time-series data through multi-sources satellite imagery modelling which combine land subsidence and urban morphology data in Jakarta, Indonesia. As one of the world’s fastest-sinking cities, Jakarta has been experiencing rapid land subsidence due to excessive groundwater extraction, rapid urbanisation and population growth [1,2]. However, official datasets are often unavailable, incomplete or based on research that are not publicly available. Beyond Jakarta, other coastal cities worldwide are experiencing similar problems, such as Ho Chi Minh City, Bangkok, Hanoi, Manila [3,4], New Orleans [5], Venice [6], and Mexico City [7] and data scarcity is often a major problem for an up to date and detailed modelling and predictions. Beyond the use of the Jakarta case, the methods can be replicated in other cities, promoting FAIR (Findable, Accessible, Interoperable, Reusable) principles [8].
• This dataset is valuable for urban planners, modellers, scholars of climate risk/adaptation/mitigation, and policymakers, particularly those working on urban resilience and risk assessment. The dataset can provide a comprehensive information to address a critical gap in understanding the complex interrelationships between human-induced activities and urban planning decisions that may contribute to and affect subsidence and subsequently, flooding processes.
• The standardised spatial resolution and temporal alignment (mostly from 2016 – 2024) make it suitable for machine learning applications, scenario simulations and predictive modelling. Making this data available would accelerate scientific understanding of urban environmental analysis and support effective adaptation and mitigation strategies in subsidence-prone and flood-risk areas globally. The methodological framework and data structure established in this dataset can serve as a replicable template for generating comparable datasets in other global sinking cities, thereby ensuring transferability, which is particularly useful in the Global South context of data-scarce cities.
• This dataset can also be used alongside other datasets, such as official census and government datasets, for example, to test urban planning interventions for sinking cities. The impact and knowledge transfer from the database system have the potential to go beyond its original geographic scope, contributing to the advancement of urban resilience planning.
• This dataset serves as an example of data modelling in which accurate, official datasets are scarce. By making the dataset publicly available, other researchers could save time and resources by reusing it. For example, the land subsidence dataset often relies on expertise in geotechnical and geomatic engineering, remote sensing, and hydrogeology. This is also similar to the urban morphology dataset. Publishing the modelled datasets enables research in other disciplines, such as urban planning and climate science, to reuse our output without rebuilding the models and integrate the data into climate modelling and simulations.

## 2 Background

Jakarta, Indonesia, is one of the world’s fastest sinking city that experiences frequent flooding throughout the different parts of the city [1,2,9]. With approximately 27 rivers traversing the city, and the low-lying topography on the coast of Java Sea, Jakarta has been historically vulnerable to flooding [10]. However, the recent decades have shown that Jakarta flooding has become more frequent and severe due to various reasons, including inadequate urban planning, rapid urbanisation, and land subsidence due to groundwater extraction with inadequate piped water provision in many parts of the city [2,11]. The theoretical urban hydrology and flood risk research have also identified land cover change, surface imperviousness, ground deformation, and residential morphology as the principal modifiable determinants of urban flood susceptibility, including in Jakarta [12,13]. Jakarta and its metro area span 4384 km^2^ with population density of 13,000 people per km^2^ [11]. As one of the densest and most populous cities in the world, Jakarta’s frequent flooding affects >10 millions of its inhabitants, with those most vulnerable placed at the forefront of the disaster risks.

Despite the growing literature, openly accessible datasets that integrate these dimensions, especially with a consistent temporal and geospatial framework, remain scarce for many rapidly urbanising regions. Previous studies remain dependent on official statistics and other data forms such as news media [10]. This dataset was compiled to address the gap by bringing together remotely sensed and vector-based spatial layers under a unified temporal and spatial framework. By making these layers available in an integrated form, modelled from Copernicus, Landsat, Sentinel-2, and building-footprint sources, the dataset enables researchers, planners, and policymakers to examine multi-driver flood risk dynamics at fine spatial and temporal scales without the need to undertake the substantial pre-processing burden involved in harmonising these products independently.

## 3 Data Description

The dataset is provided in three formats: raster (TIFF), vector (shapefile) and tabular (CSV).

**Table:** 

No	Title	Temporal Scale	Description	Data Type	Sources
1	Land Subsidence	2016–2024	Land subsidence rates.	Raster	Copernicus
2	NDVI and NDBI	2015–2024	Normalised Difference Vegetation Index (NDVI) maps showing green-covered areas and Normalised Difference Built-up Index (NDBI) maps highlighting urban areas.	Raster	Landsat
3	Impervious Surface Area	2016–2024	Spatial layer showing the extent of impervious surfaces such as roads and buildings.	Raster	Sentinel-2
4	Proportion of Residential Area	2023	Proportion of land classified as residential at the RW (neighbourhood) level.	Vector	Google Open Buildings 2.5D Temporal Dataset

## 4 Experimental Design, Materials and Methods

As the database contains several datasets, this section explains each dataset according to its (1) data source and records; (2) methods; (3) visualisation; (4) technical validation and (5) limitations.

### 4.1 Land subsidence

**Data source and records:** To analyse land subsidence, dataset from the Centre of the Observation and Modelling Earthquakes, Looking Inside the Continents from Space (COMET-LiCS) initiative, which contains unwrapped interferograms (unw) and coherence (cc) from Sentinel-1 SAR were used. This is processed end-to-end with the open-source package LiCSBAS, which efficiently processes time-series data while minimising disk space, using freely available LICSAR products [14].

**Methods:** The LiCSBAS workflow consists of two main steps: data preparation and time-series analysis.

Data Preparation: GeoTIFF files of unwrapped interferograms (unw) and coherence (cc) were downloaded from the COMET-LiCS web portal for a specified LiCSAR frame (typically 250 × 250 km), covering both ascending and descending tracks. These time-series datasets, typically available from late 2014 onward, are provided in a consistent geographic reference system. The download also includes key metadata such as line-of-sight (LOS) vectors and perpendicular baselines. As our research requires data from 2016 to 2024, 20,160,101 (January 1, 2016) and 20,241,231 (December 31, 2024) are selected as temporal boundaries for processing. The COMET-LiCS web portal provides ascending data covering the 2016 to 2024 period but lacks descending data that satisfies the defined temporal boundary, as it is only available from 2016 to 2023. The GeoTIFF files of unw and cc of DKI Jakarta (Frame ID: 098A_09673_121,312) are downloaded from the COMET-LiCS web portal.

The downloaded GeoTIFF files, unw and cc, are converted to float32 and uint8 format, respectively. The GeoTIFF files are converted without down sampling, since the data already have a spatial resolution of 100 m. Further down sampling will only reduce accuracy, since the aim is to produce a final output at 30 m resolution (achieved via kriging interpolation of the LiCSBAS output). To reduce tropospheric noise in InSAR time series, LiCSBAS supports correction using delay maps derived from the Generic Atmospheric Correction Online Service (GACOS) [10,11,12], which are based on European Centre for Medium-Range Weather Forecasts (ECMWF) data and available globally in near-real time.

**Fig. 1:**
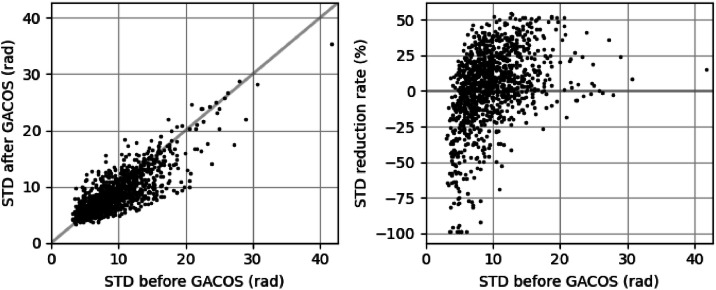
Correlation diagrams of the standard deviation (STD) of unwrapped phases in the 1203 interferograms before and after the GACOS correction. Grey lines denote no change in STD.

To assess the significance of GACOS correction, the workflow was tested with and without it, as shown in Fig. 1. The experiment revealed that GACOS correction does not yield meaningful results, as the standard deviation (STD) of unwrapped phases per interferogram increases (rather than decreases) by 2.6% on average and 5.9% at the median, indicating minimal tropospheric noise mitigation. The absolute difference in velocity between with and without the GACOS correction is also minimal, with both the mean and standard deviation of 0.2 mm, indicating that the GACOS correction can be omitted altogether. The DKI Jakarta bounding box coordinates are then used to clip the original image.

Time Series Analysis: The process begins by assessing the quality of the unwrapped interferograms using the average coherence and the percentage of valid pixels. This is followed by network refinement through loop closure using interferogram-level filtering, which avoids false corrections that pixel-level methods may introduce. LiCSBAS then performs SB (Small Baseline) inversion using the new small-baselines subset (NSBAS) method to estimate displacement time series and mean velocity from a network of unwrapped interferograms and estimates the standard deviation (STD) of the mean velocity using the percentile bootstrap method. All of these are implemented using LiCSBAS default settings [14].

**Fig. 2:**
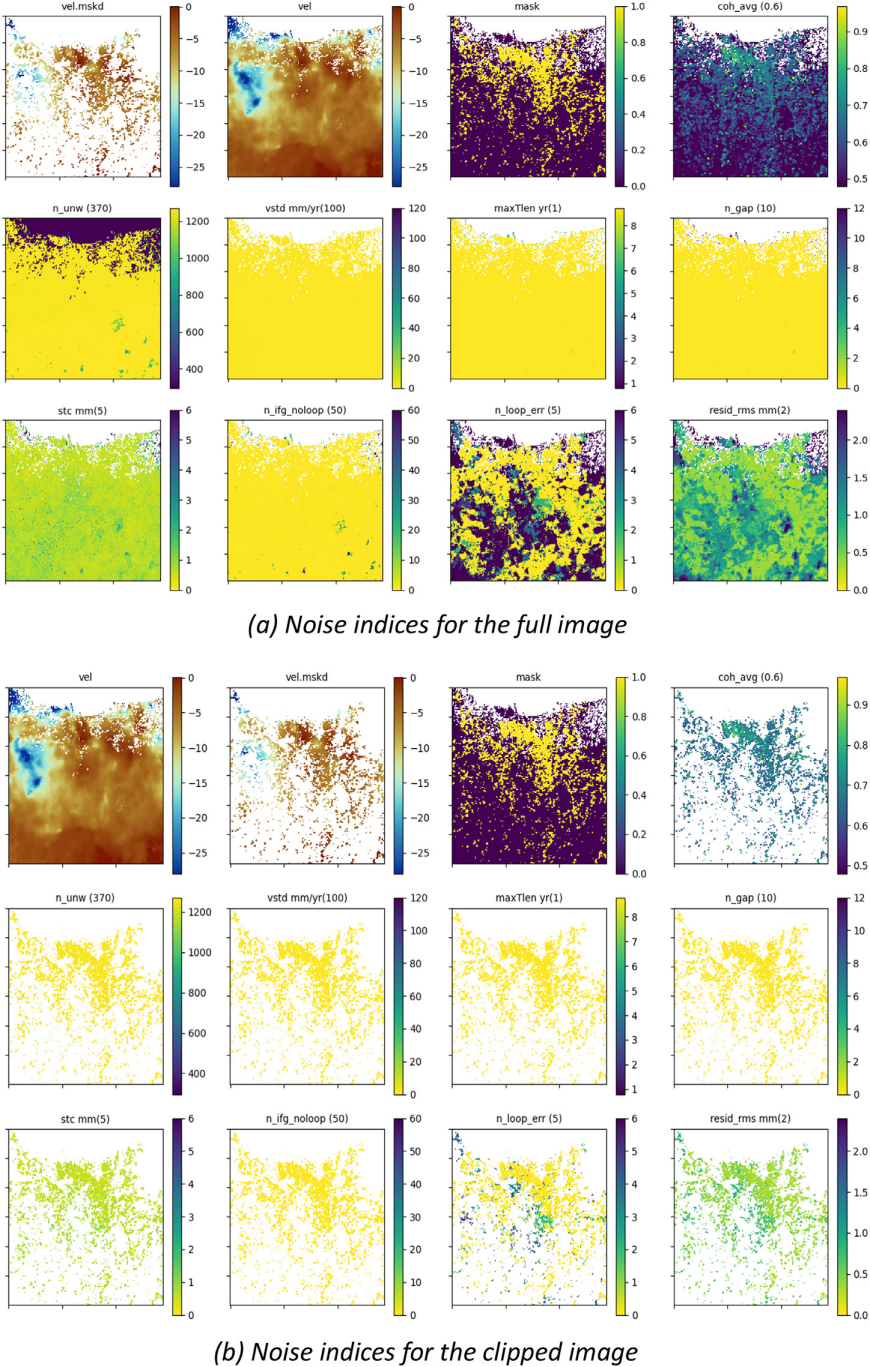
Noise indices.

To ensure that only reliable displacement time series are used, LiCSBAS masks "bad" pixels based on several noise indices (e.g., coherence, velocity standard deviation, network gaps, STC). In this processing step, noise indices are observed to determine the optimum threshold values. Coherence is a common metric for assessing pixel quality in InSAR processing, but different studies use different thresholds. The European Space Agency (ESA), producer of the Sentinel-1 SAR data used by LiCSBAS, mentions that a good coherence is >0.6 [15]. This is in agreement with our resulting noise indices, as Fig. 2a shows that areas with average coherence of <0.6 contain pixels with higher unclosed loops after network refinement (*n_loop_err*) and RMS of residuals in the SB inversion (*resid_rms_mm*). Other parameters, such as velocity STD, network gaps, etc., exhibit only a small number of low-quality pixels, which are adequately handled by their respective default threshold values, as shown in Fig. 2b Thus, the coherence threshold was set to 0.6, while all other parameters were left at their default values to ensure high-quality output pixels.

**Fig. 3:**
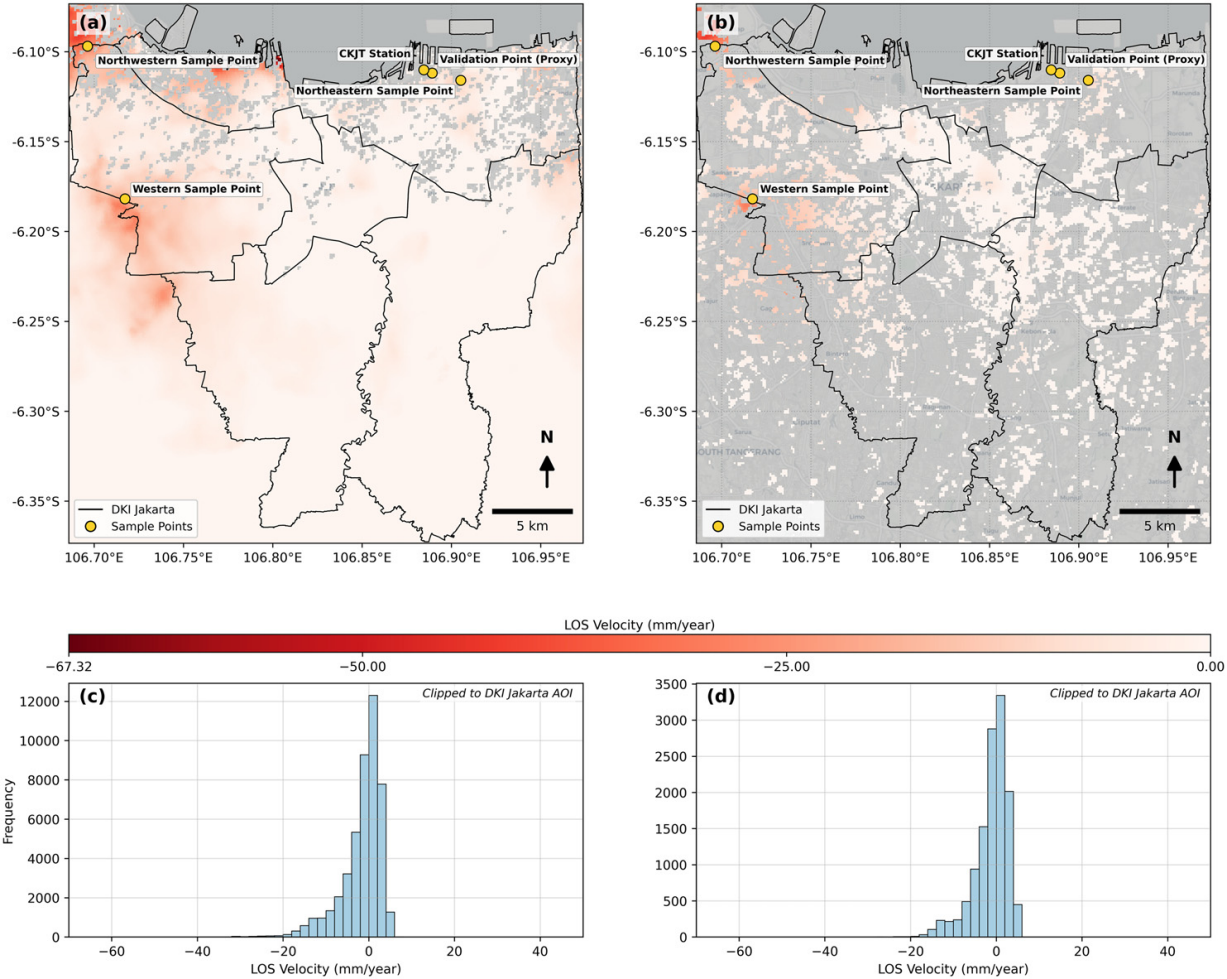
Land subsidence (left) and masked land subsidence (right) in DKI Jakarta and its histogram. Positive values are capped at 0 in both maps.

**Visualisation:** The LiCSBAS processing pipeline revealed the LOS velocity of ground deformation in DKI Jakarta at a spatial resolution of ∼100 m. The deformation is concentrated in three main regions of DKI Jakarta: the western, northwestern, and northeastern regions. The LOS velocity distribution and histogram are shown in Fig. 3; the left panel shows the land subsidence values before applying the quality mask, and the right panel shows the values after masking. The minimum value (maximum subsidence) in DKI Jakarta is −29.86 mm/year (masked). The western and northwestern hotspots are situated in a densely populated area and a coastal area (warehouses and construction area of Pantai Indah Kapuk (PIK) 2), respectively, along the border with Tangerang City, Banten Province. The northeastern region, by contrast, is located near the Port of Tanjung Priok.

**Fig. 4:**
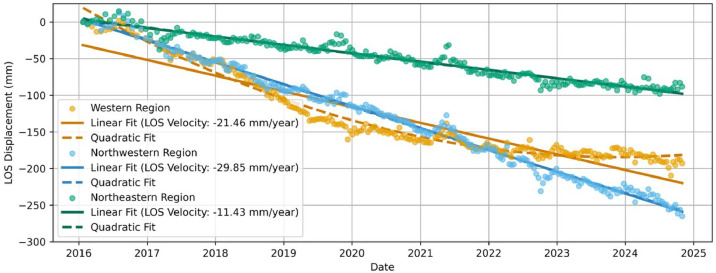
Subsidence trend for western and northwestern hotspot regions.

Subsidence from the three hotspots exhibits two distinct trends, as shown in Fig. 4. The western region has experienced rapid subsidence since 2016, which has since slowed significantly. Furthermore, the northeastern and northwestern regions are experiencing linear deformation with no significant signs of slowing, albeit at markedly different velocities.

**Fig. 5:**
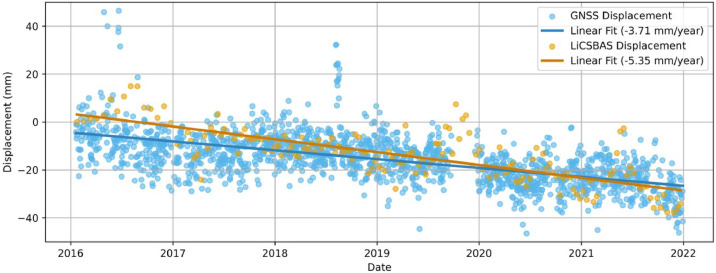
Linear fit of GNSS and LiCSBAS measurements.

**Technical Validation:** Despite InSAR providing a larger extent for land subsidence analysis, the results should always be validated against ground measurements. One such ground measurement station (the CJKT station) in DKI Jakarta is maintained by *the Badan Informasi Geospasial* (Geospatial Information Agency) of Indonesia as part of the InaCORS network of continuously operating reference stations (CORS), providing ground deformation measurements using the global navigation satellite system (GNSS). The CJKT station is in Tanjung Priok, but the LiCSBAS output contains no data points at the station’s coordinates. Therefore, although the CORS station was not directly intersected, the closest data point to the station (∼561 m away) was utilised as a proxy (Fig. 5). Susilo et al. [13] provided the station’s measurement data from 2011 to 2021. To align with both LiCSBAS and station measurements, only the 2016–2021 data were used for this validation. Additionally, data points from the station’s measurements with vertical-displacement uncertainty in the top 20th percentile were removed and only the cleaned data for our analysis were used, unless stated otherwise.

Both the LiCSBAS’s and the station’s measurements show a matching linear subsidence. To calculate velocity from both datasets, linear regression is used to estimate the slope of the best-fitting line (i.e., annual velocity) from LiCSBAS and CORS measurements. Our LOS velocity overestimates the vertical velocity derived from GNSS measurements by 1.64 mm/year, as shown in Fig. 5. To assess the precision and accuracy of the LiCSBAS results, the standard deviation and RMSE with those from the CJKT measurements were compared. The difference between the two standard deviations is 0.78 mm, with a root-mean-square error (RMSE) of 9.40 mm. Our result aligns with Harintaka et al. [14], showing that the subsidence hotspots in DKI Jakarta are located within the same three regions. The comparison between the subsidence trend from our study and that of Harintaka et al. [14] indicates that the western region trend is consistent, with subsidence slowing. Furthermore, our results show alignment in vertical displacements relative to the CJKT station measurements.

The results of the processing pipeline cannot be decomposed into LOS displacement/velocity and vertical displacement/velocity due to insufficient descending data. In a study of land subsidence in Jakarta using InSAR, Ng et al. [15] found that a horizontal movement of 10 mm/year to the east could introduce an error of 8 mm/year in the vertical direction. Both values are technically incomparable, as vertical displacement/velocity is more accurate than their LOS counterparts because they are free of horizontal movement. In addition, the validation pixel used is distant from the CJKT station, which means it does not capture displacement exactly at the area measured by the CJKT station.

**Fig. 6:**
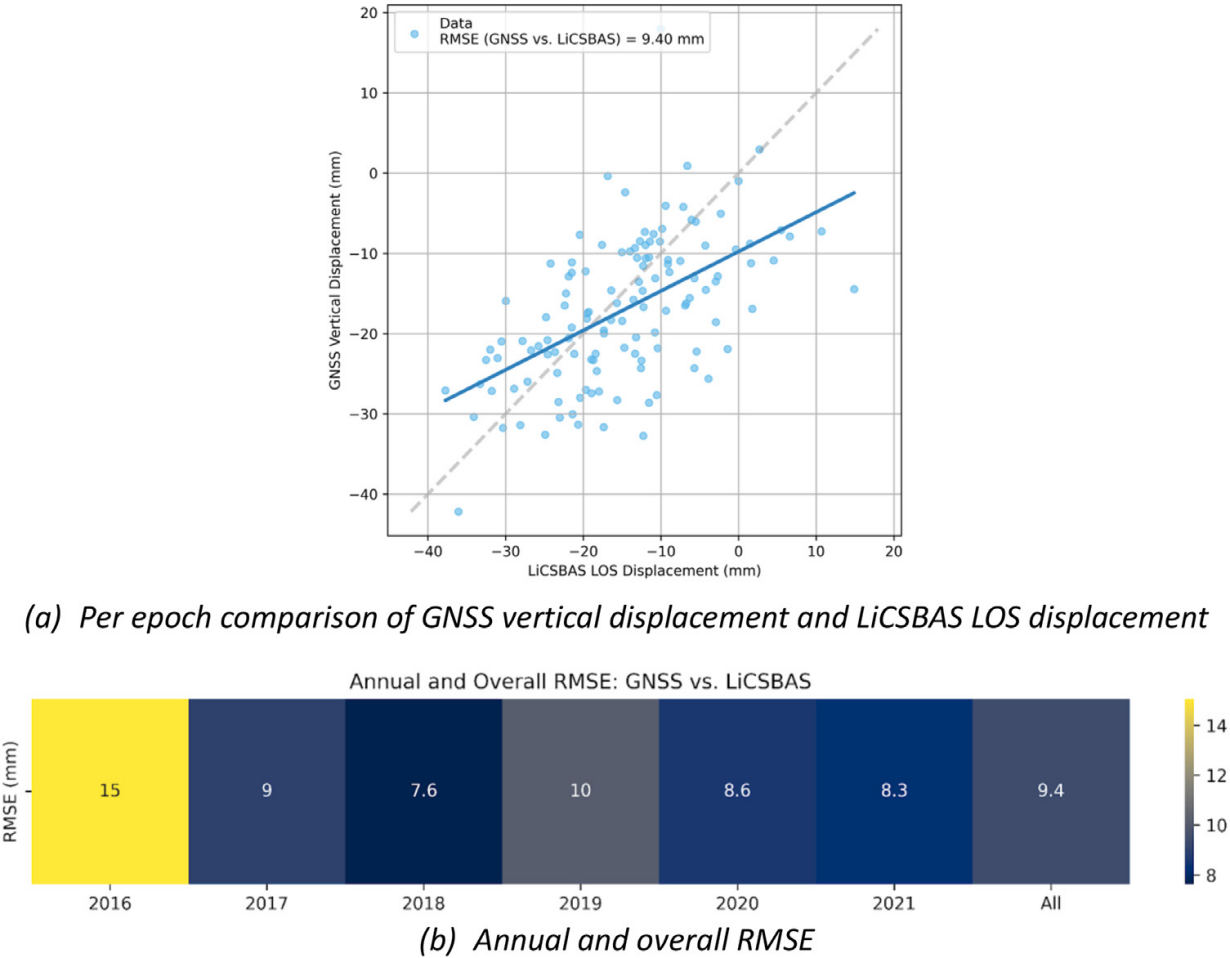
LiCSBAS against GNSS measurements.

Despite the odds against the LOS results, the precision and accuracy are good compared with GNSS measurements and another similar study. When compared to GNSS measurements epoch-to-epoch, the LiCSBAS output appears to underestimate the vertical displacement value (Fig. 6a), despite the resulting LOS velocity overestimating the vertical velocity. Compared with GNSS measurements, the precision and accuracy of our results, as measured by STD and RMSE, differ by <1 mm in STD and <1 cm in RMSE. The annual RMSE is shown in Fig. 6b The source of error is uncertain, but it may be attributed to horizontal displacement, atmospheric conditions, and orbital errors [14,16]. The subsidence trend is consistent with another similar study in a region with LiCSBAS data. This demonstrates that the LiCSBAS analysis package handled the processing effectively, even when producing only LOS displacement/velocity.

**Fig. 7:**
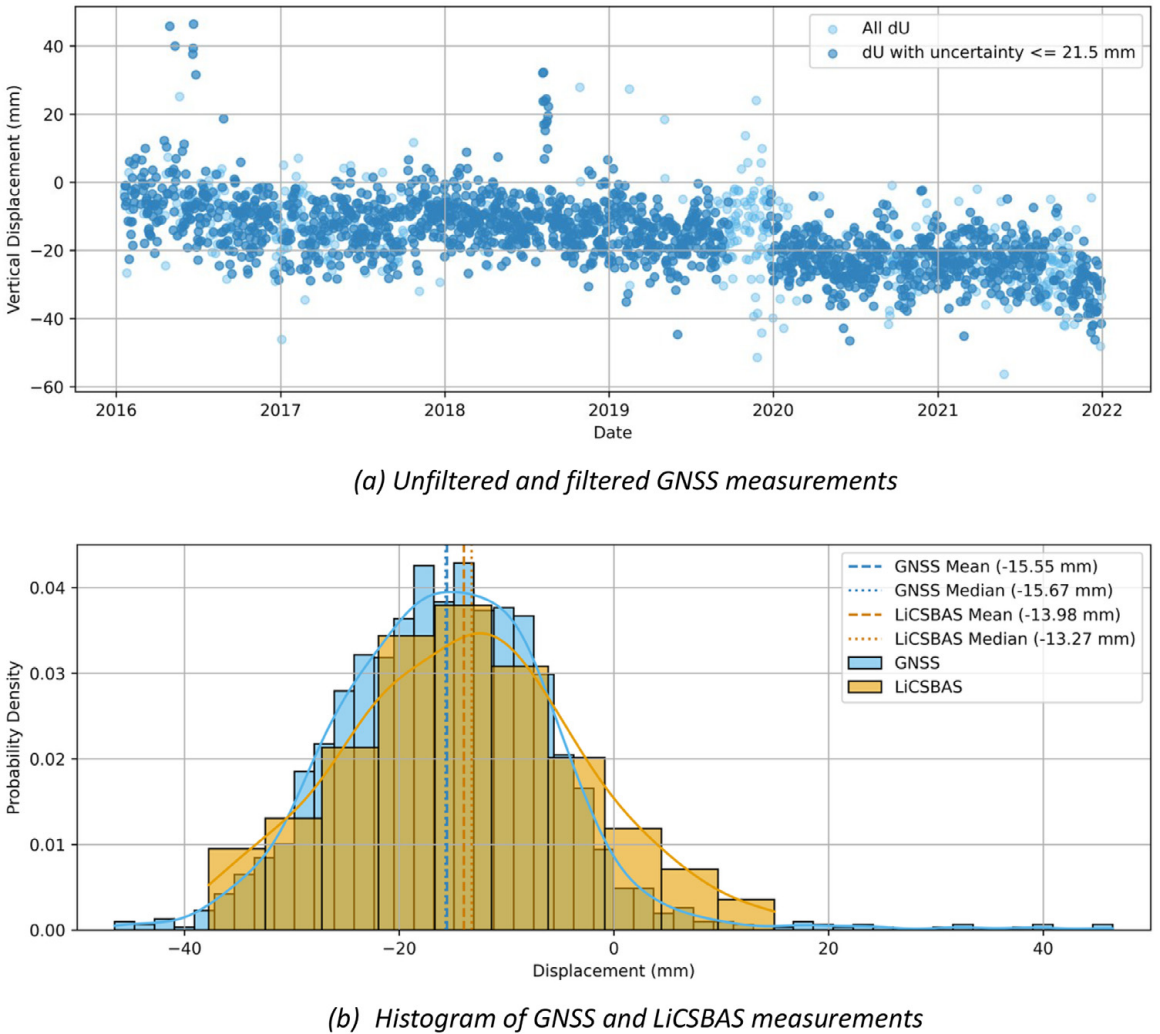
Reliability of GNSS measurement.

Even at a ground measurement station, data from the CJKT station are not error-free. For starters, the standard deviation of vertical displacement is high, at 10.23 mm. This could be attributed to the station's location, which is surrounded by tall buildings, thereby causing signal obstruction and multipath [16]. Before excluding data with vertical uncertainty exceeding the top 20%, the mean value is 18.92 mm, and the standard deviation is 6.69 mm [17]. After correction, the average and standard deviation of vertical uncertainty were significantly reduced to 16.76 mm and 2.86 mm, respectively (filtered and unfiltered data shown in Fig. 7a). It is also observed that many measurements from late 2019 exhibit the highest vertical uncertainty. Nevertheless, the LOS and vertical displacements are in overall agreement, as shown in Fig. 7.

**Limitations:** To ensure pixel reliability, a coherence threshold of 0.6 was applied, in accordance with ESA's recommendation [15]. While this threshold effectively filters out pixels with high unwrapping errors, it introduces a trade-off between data quality and spatial coverage. The northern coastal region of DKI Jakarta, characterised by vegetation, proximity to the sea, ongoing construction at PIK 2, and warehouse facilities, exhibits inherently low coherence due to temporal decorrelation. As a result, pixels in this area failed the quality filter and were excluded from the final output, as evidenced by the comparison of masked and unmasked results in Fig. 3.

The LiCSBAS processing workflow calculates all displacement values relative to a single reference pixel, selected as the pixel with the smallest RMS deviation and the fewest data gaps across the interferogram network. This approach implicitly assumes that the reference pixel experiences zero displacement throughout the study period. However, if the reference pixel is itself subsiding (or uplifting), a systematic bias propagates to all other pixels in the dataset, effectively shifting the entire displacement field by an unknown offset. In this study, no independent verification of the reference point's stability was conducted. While the selection criteria favour temporally consistent pixels, consistency does not guarantee absolute stability. Future work could address this limitation by cross-referencing the reference pixel location with independent geodetic measurements or by selecting a reference point in a geologically stable region outside the active subsidence zone.

The validation of InSAR-derived displacement against ground measurements is constrained by both national geodetic infrastructure and data availability. DKI Jakarta, despite being a megacity experiencing heterogeneous subsidence patterns, is served by only two InaCORS GNSS stations maintained by Badan Informasi Geospasial: CJKT in Tanjung Priok (Jakarta Utara) and CJKU in Kemayoran (Jakarta Pusat). This sparse coverage limits the spatial representativeness of any ground-based validation effort. Furthermore, Susilo et al. [17] published processed, analysis-ready GNSS data specifically for stations along Java's northern coast to support land subsidence research in the region. Their dataset includes CJKT but excludes CJKU, likely because CJKU's inland location falls outside their coastal focus. Consequently, validation in this study is constrained to a single station. An additional spatial mismatch arises from the absence of LiCSBAS data points at the exact CJKT coordinates, necessitating the use of a proxy pixel located approximately 561 m from the station. While this proxy captures ground behaviour in the vicinity, it does not represent identical ground conditions. Expanded CORS network coverage in DKI Jakarta, coupled with publicly available processed GNSS data, would substantially benefit future subsidence monitoring and validation efforts.

Despite these limitations, the data processing methodology has ensured that the model results remain accurate and valid for land subsidence analysis in DKI Jakarta. The LiCSBAS workflow incorporates multiple quality-control measures to filter unreliable pixels and retain only high-confidence displacement estimates, including loop-closure analysis to identify and remove interferograms with unwrapping errors, coherence thresholds aligned with ESA recommendations (>0.6), and noise masking based on velocity standard deviation and network gaps. Validation against GNSS measurements from the CJKT station demonstrates strong agreement, with a standard deviation difference of <1 mm, an overall RMSE of <1 cm, and a velocity difference of only 1.64 mm/year despite the LOS-to-vertical measurement mismatch. Furthermore, our results align with those of Harintaka et al. [14] in identifying the same three primary subsidence hotspots and matching the slowing subsidence trend in the western region. Notably, our LOS displacement trend shows better agreement with the CJKT station measurements than the ascending data from Harintaka et al. [16] as evidenced by the temporal agreement in Fig. 5, in contrast to the disagreement reported in their study. These validation outcomes confirm that the processing pipeline has effectively mitigated the constraints inherent to LOS-only analysis and limited spatial validation, producing subsidence estimates that are both precise and accurate for characterising ground deformation in the study area.

### 4.2 NDVI and NDBI

**Data source and records:** To analyse the Vegetation and Built-up index for spatial-temporal records, the datasets were officially sourced from the U.S. Geological Survey (USGS) in Collaboration with NASA. Multi-temporal Landsat imagery was acquired for benchmark years 1990, 2000, 2010, 2024 using Landsat 5 (1984–2013), Landsat 8 (2013-present), and Landsat 9 (2021-present) [18,19], however only data from 2015–2024 was included in the repository.

**Fig. 8:**
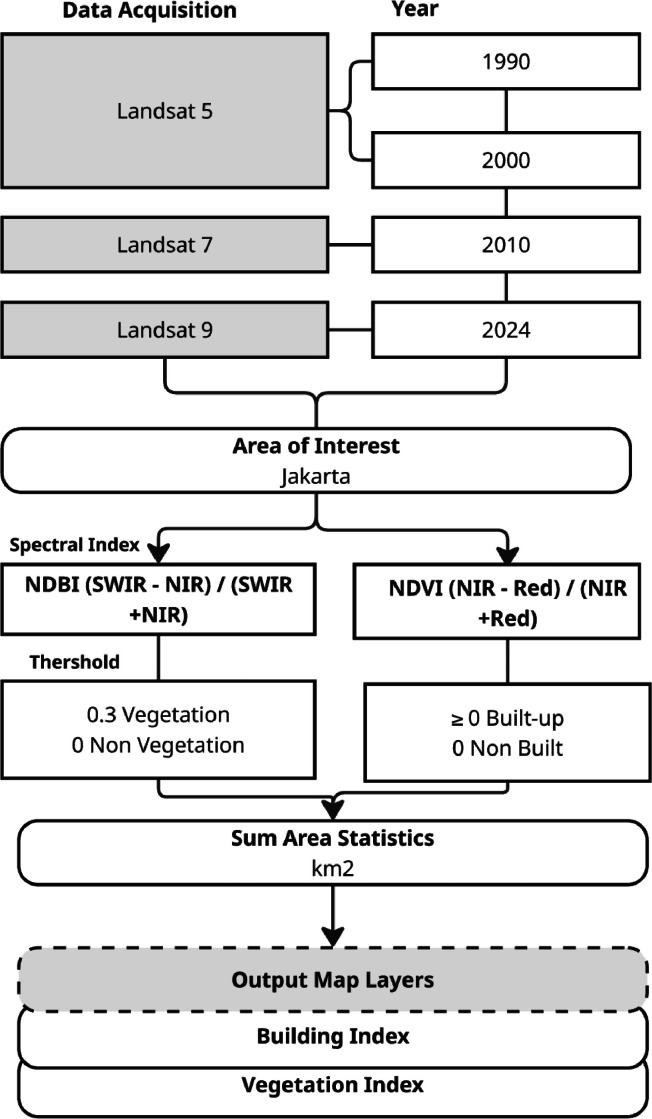
NDBI and NDVI methodology.

**Methods:** The Normalised Difference Built-up Index (NDBI) and the Normalised Difference Vegetation Index (NDVI) are widely used spectral indices for interpreting land surface changes in multispectral satellite imagery. NDBI is primarily used to identify and track changes in urban and built-up areas, as it highlights surfaces associated with buildings and infrastructure [20]. Conversely, NDVI is used to capture changes in vegetation cover and greenness, reflecting variations in plant density and reflectance of healthy vegetation [21]. (1), (2) describes how NDBI and NDVI are computed, and Fig. 8 shows the methodological flow. Together, these indices provide complementary perspectives on the spatial dynamics of urban expansion and vegetation change over time.

(1) $$\begin{array}{c} \mathrm{NDBI}=\left(\mathrm{SWIR}-\mathrm{NIR}\right)/\left(\mathrm{SWIR}+\mathrm{NIR}\right)\\ \text{SWIR}=\text{Shortwave}\;\text{infrared}\;\text{band}\\ \text{NIR}=\text{Pixel}\;\text{values}\;\text{from}\;\text{the}\;\text{near}-\text{infrared}\;\text{band} \end{array}$$

(2) $$\begin{array}{c} \mathrm{NDVI}=\left(\left(\mathrm{NIR}-\mathrm{Red}\right)/\left(\mathrm{NIR}+\mathrm{Red}\right)\right)\\ \text{NIR}=\text{Pixel}\;\text{values}\;\text{from}\;\text{the}\;\text{near}-\text{infrared}\;\text{band}\\ \text{Red}=\text{Pixel}\;\text{values}\;\text{from}\;\text{the}\;\text{red}\;\text{band} \end{array}$$

**Data Preparation:** Built-up and non-built-up areas in DKI Jakarta using a multi-temporal Normalised Difference Built-up Index (NDBI) and approach derived from Landsat 5, Landsat 7, and Landsat 9 imagery.

The analysis begins with defining the region of interest (ROI) corresponding to the Jakarta administrative boundary. Multi-year satellite data are then acquired for representative benchmarks in 1990, 2000, 2010, and 2024 to capture long-term urban growth dynamics.

NDBI values are subsequently classified into two land-cover classes using a threshold-based rule: NDBI ≥ 0 indicates built-up areas, while NDBI < 0 represents non-built surfaces such as vegetation, soil, and water bodies. On the other hand, NDVI values are subsequently classified into two land-cover classes using a threshold-based rule: NDVI 0 indicates vegetation, whereas NDVI 1 indicates non-vegetation, such as built-up areas. The classified rasters are converted to pixel-area statistics to quantify the total built and non-built extents in square kilometres, and the results are exported as GeoTIFF map layers.

**Fig. 9:**
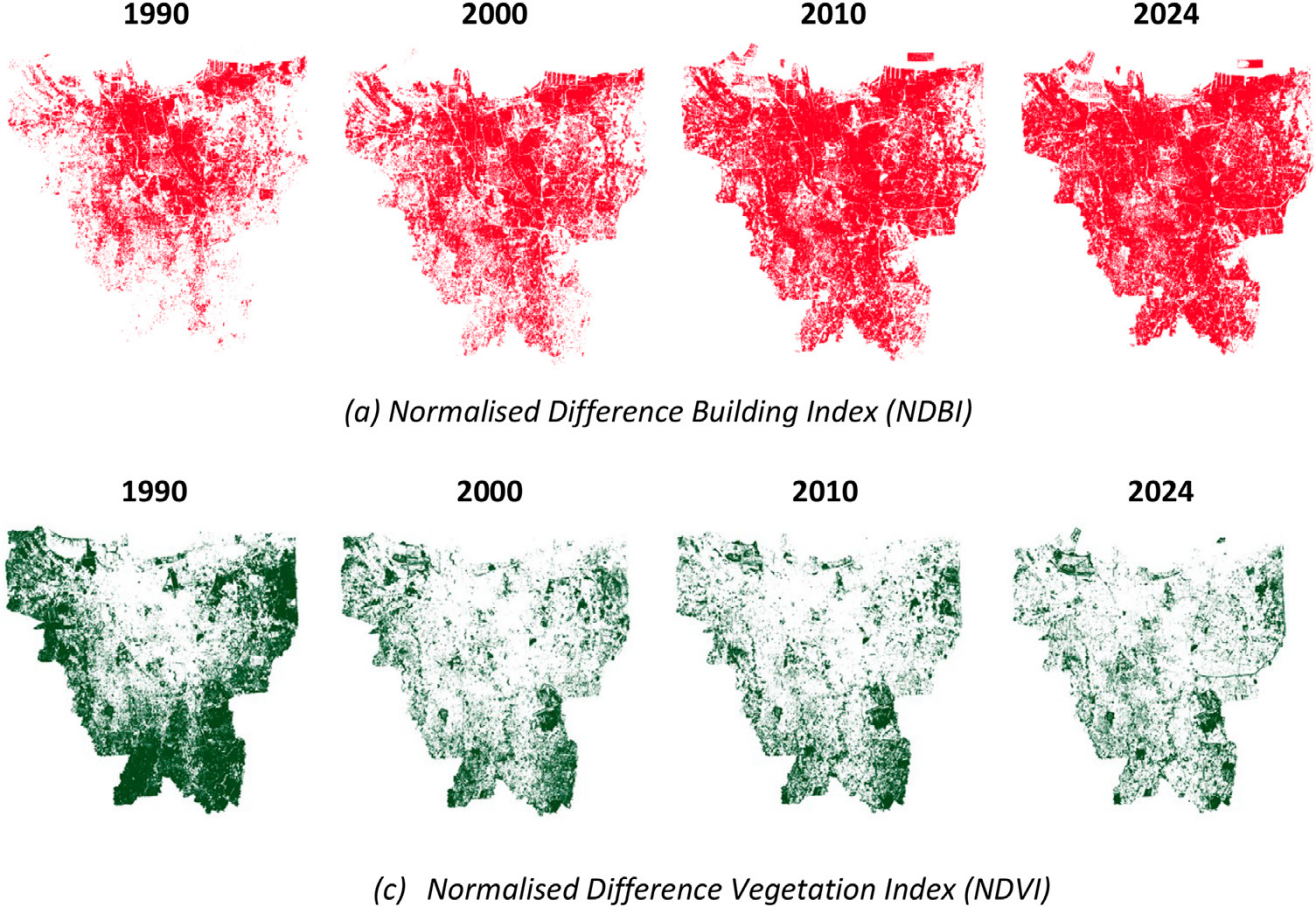
NDBI and NDVI visualisations.

**Visualisations:** The integrated NDVI and NDBI analysis indicates a pronounced land-cover transformation driven by urban expansion between 1990 and 2024. Built-up areas increased steadily from 148.02 km² in 1990 to 251.79 km² in 2000, 306.55 km² in 2010, and 337.33 km² in 2024, reflecting sustained urban growth, densification, and spatial consolidation as can be seen in Fig. 9. In contrast, vegetated areas declined sharply from 420.37 km² in 1990 to 299.28 km² in 2000, reaching a minimum of 282.56 km² in 2010, indicating significant conversion of green and open land to urban uses during periods of rapid development.

**Technical Validation:** The validation of the NDBI and NDVI-derived results was conducted through visual comparison with high-resolution reference imagery, including recent Google Maps/Google Satellite imagery. The spatial patterns of identified built-up and vegetated areas were cross-checked against contemporaneous optical imagery to assess the consistency of land-cover representation. This qualitative visual validation approach is commonly adopted in urban and land-cover studies, where detailed ground truthing using higher-resolution images and comparisons between results are used to validate urban and vegetation areas.

**Limitations:** Despite their wide applicability, the NDBI and NDVI-based approach have several limitations. First, spectral confusion may occur between built-up areas and bare or sparsely vegetated land, as both surfaces can exhibit similar reflectance characteristics in the NIR and SWIR bands, potentially leading to misclassification. Second, the use of medium-spatial-resolution imagery, such as 30-metre Landsat data, limits the ability to capture fine-scale urban features, including narrow roads and small buildings, particularly in heterogeneous or high-density urban environments. Third, the validation method relies on visual interpretation of Google Satellite imagery, which is qualitative and may introduce subjectivity, particularly when temporal mismatches exist between the satellite acquisition date and the reference imagery. Despite the limitations, the method is proven useful in understanding urban development and expansion in cities.

### 4.3 Impervious surface area

**Data source and records:** Impervious surface area was derived using Sentinel-2 Level-2A Harmonised Surface Reflectance^1^ data accessed via the Google Earth Engine (GEE) platform. Sentinel-2 provides multispectral imagery at 10-metre spatial resolution for bands relevant to the Enhanced Normalised Difference Impervious Surface Index (ENDISI) formulation. Annual image composites were generated for the period 2016–2024, covering the Jakarta metropolitan area.

**Methods:** Impervious surfaces were mapped using an adaptation of the ENDISI, designed to improve spectral separability between impervious and non-impervious surfaces in urban landscapes [22]. ENDISI integrates spectral responses from visible and shortwave infrared (SWIR) bands with a context-specific correction factor that accounts for local scene conditions. Sentinel-2 bands used in the index include Band 2 (Blue, 10 m), Band 3 (Green, 10 m), Band 11 (SWIR1, 20 m), and Band 12 (SWIR2, 20 m). The index enhances the differences between impervious materials (e.g., concrete, asphalt) and pervious surfaces (e.g., vegetation, soil), supporting robust delineation of sealed surfaces.

Sentinel-2 imagery for each year was filtered to the area of interest (AOI) corresponding to the Jakarta administrative boundary, the temporal window (January 1 – December 31) for that year from 2016 to 2024 and masked for clouds and cirrus using the QA60 band. A median composite was generated annually to minimise the influence of residual clouds and sensor noise. SWIR bands were consistently resampled to 10 m by the GEE processing framework to align with visible bands. The spectral components required for the ENDISI calculation, such as MNDWI (Modified Normalised Difference Water Index), SWIR ratio, and squared MNDWI, were derived according to the index formulation, as shown in Eq. (3).

(3) $$\begin{array}{c} \mathrm{MNDWI}=\left(\mathrm{Green}-\mathrm{SWIR}1\right)/\left(\mathrm{Green}+\mathrm{SWIR}1\right)\\ \mathrm{SWIR}\;\mathrm{ratio}=\mathrm{SWIR}1/\mathrm{SWIR}2 \end{array}$$

A β correction factor was computed from scene-wide mean reflectance values to normalise the index for local conditions. Finally, the continuous ENDISI raster was constrained to the range [−1, +1] to prevent extreme spectral outliers.

(4) $$\mathrm{ENDISI}=\left[\mathrm{Blue}+\beta \left(\mathrm{SWIR}\;\mathrm{ratio}+\mathrm{MNDW}I^{2}\right)\right]/\left[\mathrm{Blue}-\beta \left(\mathrm{SWIR}\;\mathrm{ratio}+\mathrm{MNDWI}2\right)\right]$$

Binary impervious surface maps were produced by applying a threshold of −0.375 to the ENDISI raster, which was defined based on a visual comparison with higher-resolution satellite imagery in several areas. Pixels with ENDISI values above the threshold were classified as impervious surface area (ISA), and those below as pervious or non-impervious

**Visualisation:** The output comprises annual raster datasets of binary impervious and pervious surface areas at 10 m spatial resolution for Jakarta from 2016 to 2024, derived using a binary ENDISI classification. Across all years, the total mapped area remained constant at 1064.68 km², reflecting a stable spatial extent of analysis.

**Fig. 10:**
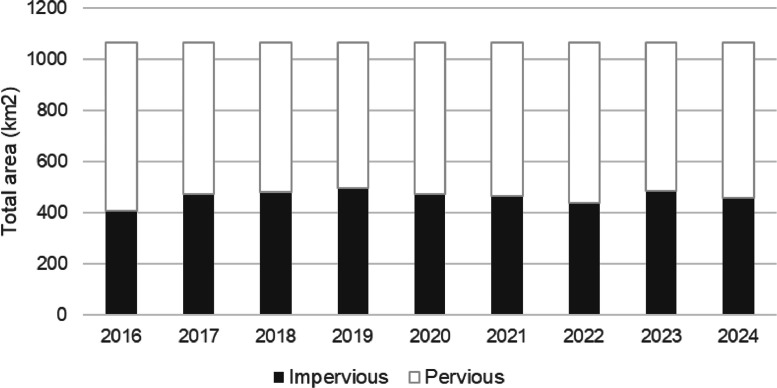
Impervious surface area (ISA) time-series comparison from 2016 to 2024.

**Fig. 11:**
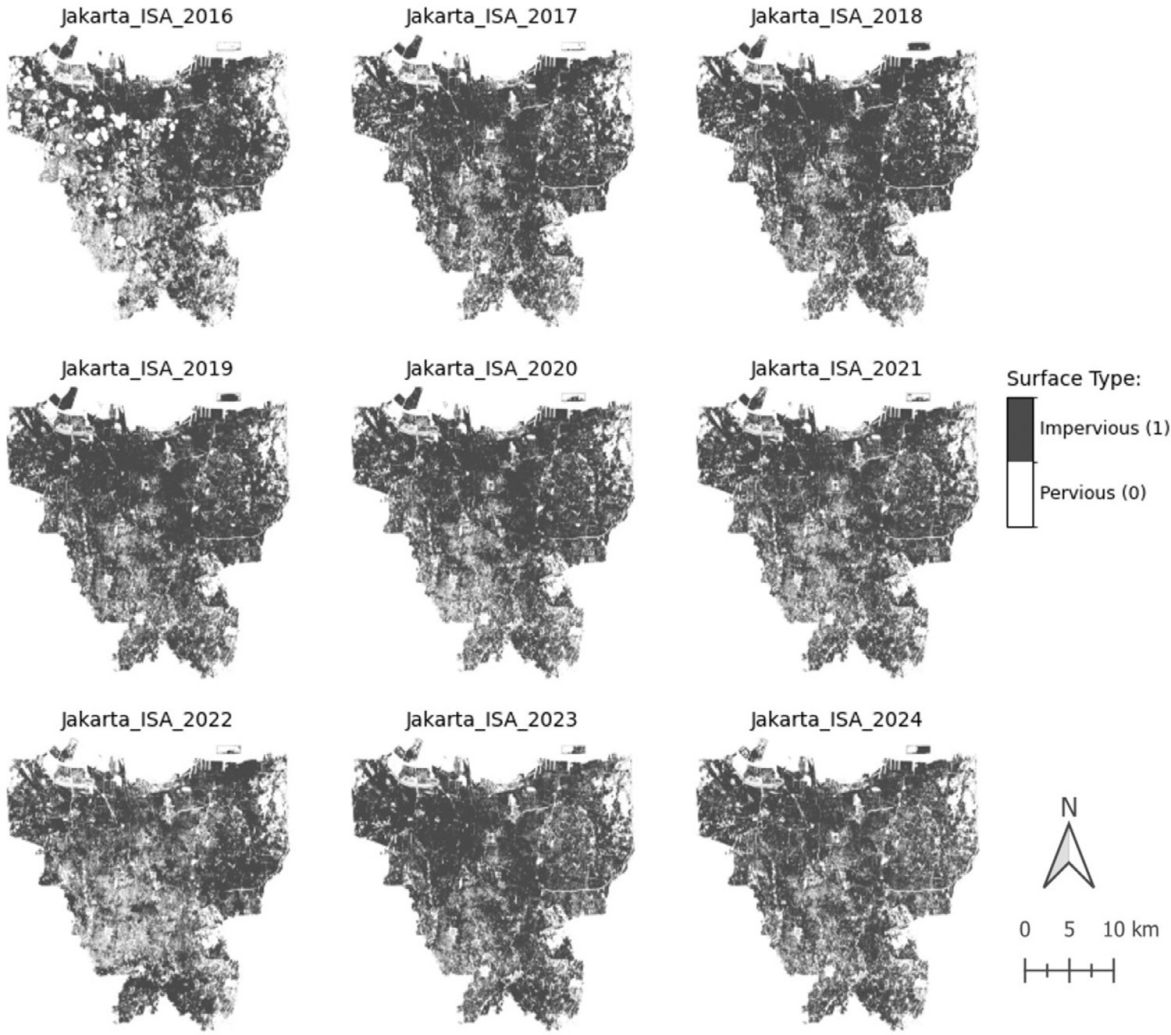
Impervious surface area (ISA) maps comparison from 2016 to 2024.

Fig. 10, Fig. 11 show that impervious surfaces consistently covered >400 km² of the study area across all years, accounting for approximately 40–50% of the total mapped area. Impervious surface extent increased gradually during the early part of the time series, reaching a relative maximum around 2018–2019, followed by a noticeable decline during 2020–2022. An increase was observed again in 2023, with a slight reduction in 2024. Conversely, pervious surface area exhibited an inverse temporal pattern. These results indicate moderate interannual variability in surface sealing across Jakarta, while overall imperviousness remains consistently high throughout the study period.

The maps in Fig. 11 capture the spatial extent and temporal dynamics of sealed surfaces across built environments, including roads, rooftops, and other anthropogenic infrastructure. The binary impervious and pervious surface layers serve as the primary dataset for subsequent spatial analysis and comparison with other thematic layers, including residential typology, NDVI-derived greenness, and flooding exposure.

**Technical Validation:** Validation of the impervious surface area (ISA) maps combined visual interpretation and cross-dataset consistency analysis. Representative locations across Jakarta were inspected using high-resolution satellite imagery (e.g., Google Satellite) to verify the delineation of roads, dense built-up blocks, and vegetated areas. This approach follows established remote sensing validation practices in data-limited urban contexts. Consistency checks were conducted using NDVI and NDBI layers. ISA showed the expected inverse relationship with NDVI and positive association with NDBI, supporting the internal consistency and thematic reliability of the impervious surface classification.

At the temporal scale, the observed increase in impervious surface extent between 2016 and 2019 is consistent with documented land conversion trends in Greater Jakarta reported by Budi et al. [23], who identified substantial growth in impervious land across Jakarta’s metropolitan region during 2017–2020. The moderate fluctuations observed during 2020–2022 may reflect temporary slowdowns in urban development. Methodologically, the use of Sentinel-2 multispectral imagery at 10 m spatial resolution aligns with previous research demonstrating its suitability for impervious surface extraction in heterogeneous urban environments [24]. These studies report reliable performance of Sentinel-2–based approaches for mapping built-up and sealed surfaces, supporting the technical robustness of the ENDISI-based framework applied in this study.

**Limitations:** Several limitations should be considered when interpreting impervious and pervious surface-area datasets. The use of a globally applied ENDISI threshold ensures temporal consistency but may not fully capture local spectral variability in heterogeneous urban environments, particularly in areas with mixed or transitional land cover. In addition, the ENDISI calculation integrates spectral bands with different native spatial resolutions, including SWIR bands originally available at 20 m and resampled to 10 m. This resampling may limit the detection of narrow features such as small lanes or thin impervious strips, particularly in dense kampung environments.

Optical data availability varies across years and locations, particularly due to cloud contamination. In the early years, particularly in 2016, persistent cloud cover led to limited cloud-free observations despite annual compositing. Residual cloud and shadow effects may therefore affect classification quality in specific areas. The validation approach, based on visual interpretation and cross-dataset consistency, does not constitute a formal quantitative accuracy assessment against independent ground truth, which remains a target for future work. Users should interpret the ISA layers as representations of relative impervious surface extent, suitable for comparative and temporal analyses, rather than as definitive per-pixel land-cover labels.

### 4.4 Proportion of residential area

**Data source and records:** The proportion of the residential area dataset for 2023 was derived from four spatial data sources. Building height information was derived from the Open Buildings 2.5D Temporal Dataset^2^ for 2023 [25]. Residential block boundaries were defined using RW (*Rukun Warga*) administrative units. Vegetation information used to approximate green space, and farmland was derived from Landsat 9 imagery via the normalised difference vegetation index (NDVI), as explained in section 4.2.

**Fig. 12:**
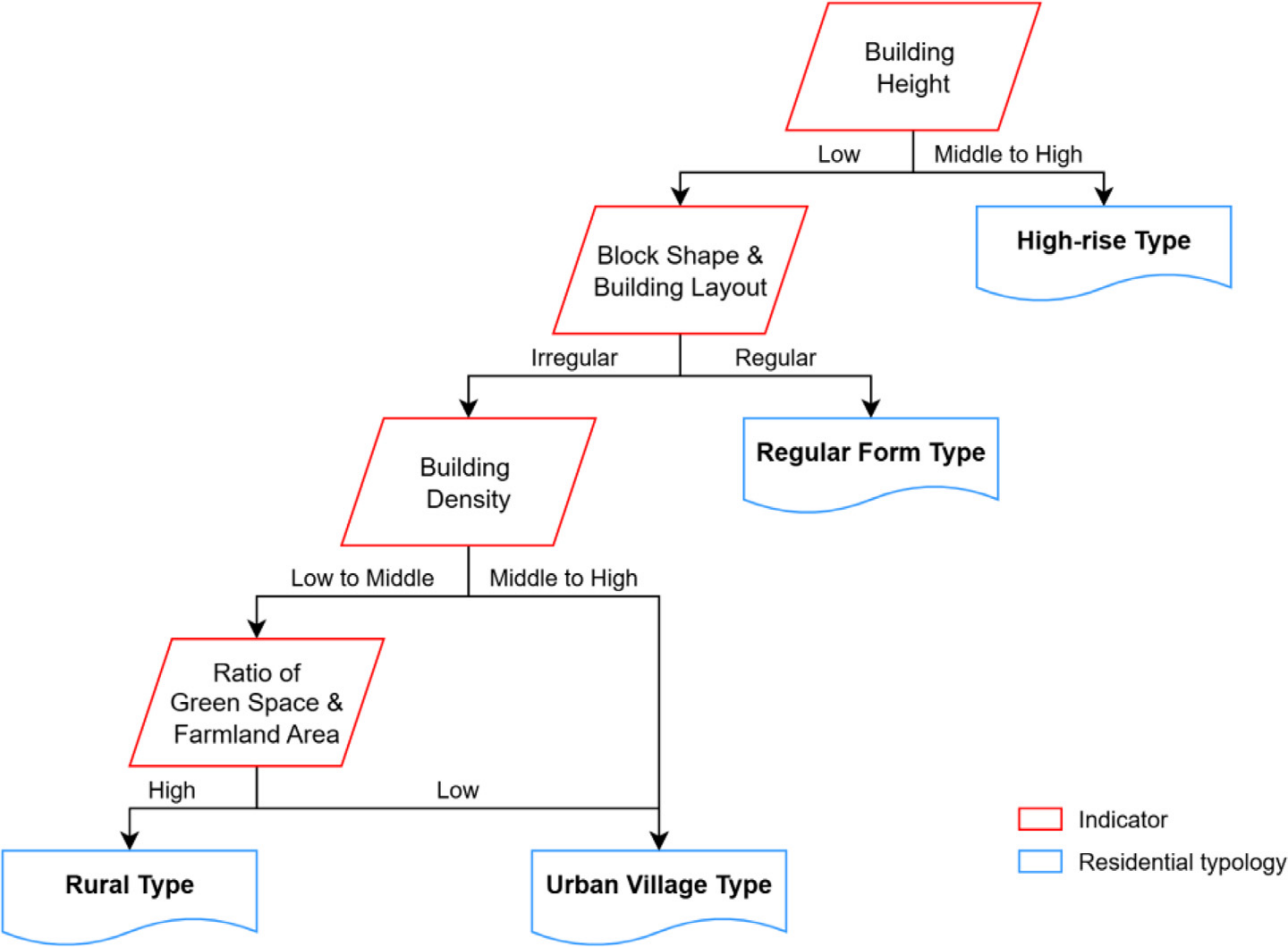
Four types of residential areas and their classification criteria (Adapted from Hayashi et al. [21]).

**Methods:** The residential area classification framework follows Hayashi et al. [26], which defines four residential typologies: (1) high-rise, (2) regular, (3) urban village, and (4) rural. Urban villages and rural areas are collectively considered as kampung settlements. Classification was performed at the RW level using four indicators: (a) building height, (b) block shape and building layout, (c) building density, and (d) ratio of green space and farmland area, as shown in Fig. 12.

Building height was converted from metres to floors and classified according to CTBUH (Council on Tall Buildings and Urban Habitat) criteria^3^ for defining high-rise buildings [27]. Despite its relative terms, a 14-floor building is categorised as a tall building, which is defined as 52–55 m for mixed-use residential, office, and hotel use. Thus, a building taller than 50 m is classified as a high-rise. All building footprints are aggregated within each residential block unit, using the majority class to determine which blocks are categorised as high-rise residential.

Block shape and building layout were quantified using geometric indicators, including building orientation, building compactness, inter-building distances, and shape index [28]. These indicators were classified using binary K-means clustering (k = 2) to distinguish between regular and irregular residential forms. Binary K-means clustering was applied to partition each indicator into two internally homogeneous groups, consistent with the dichotomous decision structure required for differentiating residential typologies [29]. As an unsupervised classification approach, K-means groups observations based on similarity in feature space without predefined labels and has been widely applied in remote sensing and spatial analysis to identify natural groupings within multivariate datasets [23].

Building density was calculated as the sum of building-area density and building-count density. Building area density is defined as the ratio of total building area to total area within a residential block, whereas building count density is defined as the number of building footprints per hectare [28]. A binary K-means clustering is used to categorise residential blocks as either middle-to-high or low-to-middle. The middle-to-high class is associated with the urban village residential type.

The ratio of green space to farmland area was approximated using NDVI as a proxy for greenness [30,31] sampled at each building centroid and classified via binary K-means clustering. All building-level classifications were aggregated to the RW level using a majority-rule approach to determine the dominant residential typology for each block. The high class is defined as rural, whereas the low class is assigned to urban villages despite having a low-to-middle class of building density.

**Fig. 13:**
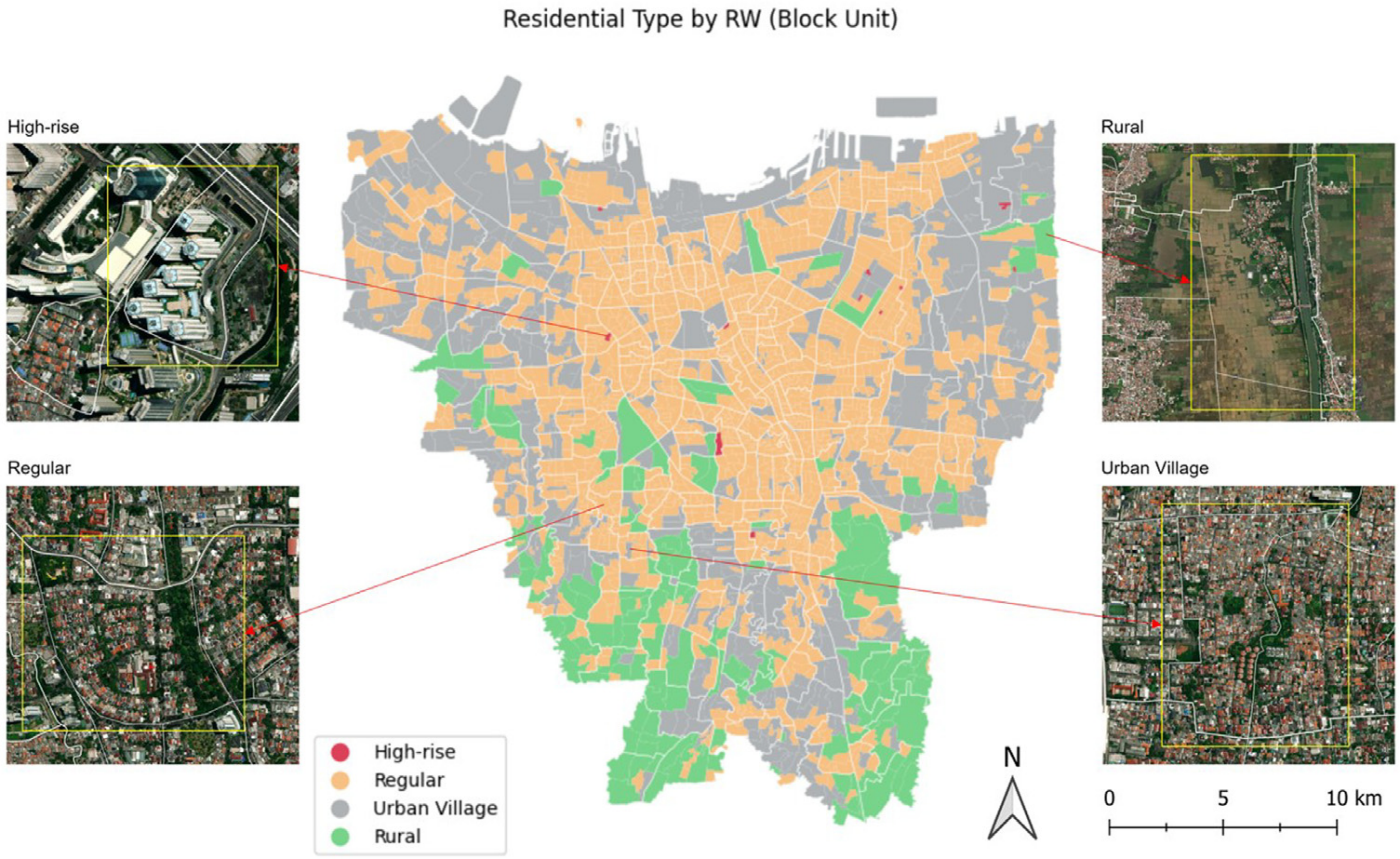
Four types of residential areas for each RW administrative unit.

**Visualisation:** The output is a vector dataset at the RW level representing the dominant residential typology for each residential block in Jakarta for 2023. Based on the classification results, a total of 2733 RW units were identified as predominantly residential. Of these, 2077 RW units were classified as regular residential areas, 493 as urban villages, 152 as rural, and only 11 as high-rise residential areas, as shown in Fig. 13.

This distribution highlights the dominance of regular residential morphology across Jakarta, with urban village and rural typologies forming substantial secondary categories, and high-rise residential areas representing a limited but distinct subset. The dataset enables spatial analysis of residential morphology and supports comparison between formal and informal settlement structures.

**Technical Validation:** Spatial validation was conducted through visual interpretation of representative RW units using recent high-resolution Google satellite imagery, focusing on building arrangement, street configuration, building density, and apparent vertical structure. This reference-image comparison approach is consistent with validation practices in urban land-cover and land-use studies, in which higher-resolution imagery is used to assess the spatial plausibility of classification outputs [32]. The mapped residential typologies exhibited spatial patterns consistent with known urban structures in Jakarta, including concentrations of high-rise residential areas in central business districts, urban village typologies in kampung-dominated neighbourhoods, and rural residential types in peripheral areas.

Additional validation was performed through cross-variable consistency checks using independently derived spatial datasets included in this study, specifically NDVI-based greenness and impervious surface area. The residential typologies demonstrated expected directional relationships: high-rise and regular residential areas were associated with higher impervious surface fractions and lower greenness, whereas rural residential areas exhibited higher NDVI values. Such internal consistency across independent spatial indicators aligns with comparative validation approaches used to assess thematic coherence in land-cover and urban mapping products [33].

**Limitations:** The residential typologies are derived from morphological and spectral proxy indicators rather than from direct land-use or socioeconomic data. Consequently, the classification captures the dominant physical characteristics of residential areas at the RW level and may not account for mixed-use functions or intra-block heterogeneity. The use of a majority-rule aggregation may oversimplify the representation of RW units containing multiple residential forms. Estimating green space and farmland relies on NDVI-based greenness as a proxy, which is subject to spectral ambiguity. Built-up surfaces, bare soil, and sparsely vegetated land can exhibit similar spectral responses, potentially obscuring the distinction between urban villages and rural residential areas. In addition, the use of 30-metre-resolution Landsat imagery limits the detection of fine-scale vegetation patterns in dense urban environments, particularly in *kampung* settlements. In addition, building height was extracted from raster data using a centroid-based sampling approach, whereby a single height value is assigned to each building footprint based on its centroid location. This approach may not fully capture height variability within large or irregularly shaped buildings and is sensitive to raster resolution and pixel alignment. Consequently, localised misclassification may occur in high-rise residential areas, particularly in mixed-use or heterogeneous urban blocks.

On the other hand, block shape, building layout, and building density indicators were classified using unsupervised K-means clustering, which is sensitive to feature scaling, initialisation, and the assumption of binary class separability. While clustering provides a consistent, data-driven classification, boundaries between residential types may be sensitive to clustering parameters and may vary when alternative configurations are used. The residential typology framework is implemented using unsupervised clustering techniques and has not been extensively validated using ground-based reference data. While the framework is grounded in established literature, classification outcomes may be sensitive to indicator selection, clustering parameters, and spatial aggregation scale.

## Limitations

Limitations related to the datasets have been included in the previous section. Below is the summary of the limitations.

**Land Subsidence.** The land subsidence dataset is constrained by limitations in pixel-quality filtering, reference-pixel stability, and ground-based validation infrastructure. Applying a coherence threshold of 0.6, in line with ESA recommendations, improves pixel reliability but introduces a trade-off with spatial coverage, particularly in the northern coastal areas of DKI Jakarta, where vegetation and proximity to the sea, among others, cause inherently low coherence and the exclusion of these pixels from the final output. The LiCSBAS workflow computes displacement values relative to a single reference pixel selected based on temporal consistency, which does not guarantee absolute stability, so any unrecognised motion at the reference point would propagate as a systematic offset throughout the dataset. Validation is further restricted by sparse national geodetic infrastructure, as DKI Jakarta is served by only two InaCORS GNSS stations, and the publicly available processed GNSS data used for validation include only the CJKT station, requiring the use of a proxy pixel located approximately 561 m away that does not represent identical ground conditions. In addition, insufficient descending data prevented the decomposition of LOS measurements into purely vertical displacement, limiting direct comparability with GNSS vertical observations.

**NDVI and NDBI.** The use of medium-spatial-resolution imageries and threshold-based rules limit the accuracy for fine scale urban features. Validation also remains qualitative, based on visual representation of an expert.

**Impervious Surface Area.** The impervious surface area dataset is subject to constraints arising from both spectral and data-quality factors. The uniform ENDISI threshold applied across all years promotes temporal consistency but may not fully account for local spectral variability in heterogeneous urban environments or transitional land-cover zones. The SWIR bands, natively at 20-metre resolution, were resampled to 10 m during processing, which may limit the accurate delineation of narrow impervious features such as small alleys in dense *kampung* areas. Persistent cloud cover, particularly in 2016, reduced the availability of cloud-free observations, and residual cloud or shadow effects may affect classification quality in specific locations despite annual median compositing. Validation remains qualitative, based on visual interpretation and cross-dataset consistency checks rather than formal quantitative accuracy assessment against independent ground truth.

**Proportion of Residential Area.** The residential typology dataset is derived from morphological and spectral proxy indicators rather than direct land-use or socioeconomic records, meaning it captures dominant physical characteristics at the RW level and may not reflect mixed-use functions or intra-block heterogeneity. Aggregation to the RW level using a majority-rule approach may oversimplify residential blocks containing multiple typologies. NDVI-based greenness used to approximate green space is subject to spectral ambiguity, and the 30-metre Landsat resolution limits fine-scale vegetation detection in dense urban settings, potentially obscuring the distinction between urban village and rural residential types. Building height estimates derived from centroid-based raster sampling may not capture within-footprint height variability, risking localised misclassification in mixed-use or large irregular buildings. Additionally, the K-means clustering approach used to classify block shape, building layout, and density indicators is sensitive to feature scaling, initialisation, and the assumption of binary separability, and the overall typology framework has not been validated against ground-based reference data.

## Ethics Statement

I confirm that the authors have read and follow the ethical requirements for publication in Data in Brief and that the current work does not involve human subjects, animal experiments, or any data collected from social media platforms.

## Credit Author Statement

**Zahratu Shabrina:** Conceptualisation, Methodology, Investigation, Resources, Data Curation, Writing – Original Draft, Writing – Review and Editing, Supervision, Funding Acquisition. **Fajrun Wahidil Muharram:** Methodology, Software, Validation, Formal Analysis, Writing – Original Draft, Visualisation. **Muhammad Asa:** Methodology, Software, Validation, Formal Analysis, Writing – Original Draft, Visualisation. **Dekka Dhirgantara Putra:** Methodology, Software, Validation, Formal Analysis, Writing – Original Draft, Visualisation.

## Data Availability

ZenodoSinking City: Multidimensional Datasets for Modelling the Sinking Jakarta (Original data).

## Footnotes

1 https://developers.google.com/earth-engine/datasets/catalog/COPERNICUS_S2_SR_HARMONIZED.

2 https://sites.research.google/gr/open-buildings/temporal/.

3 https://www.skyscrapercenter.com/criteria.
